# Simulation of fertilizer requirement for irrigated wheat in Eastern India using the QUEFTS model

**DOI:** 10.1100/tsw.2006.43

**Published:** 2006-02-22

**Authors:** Debtanu Maiti, D.K. Das, H. Pathak

**Affiliations:** ^1^ Department of Agricultural Chemistry and Soil Science, Faculty of Agriculture, Bidhan Chandra Krishi Viswavidyalaya, Mohanpur, Nadia-741252, West Bengal, India; ^2^ Unit of Simulation and Informatics, Indian Agricultural Research Institute, New Delhi - 110012, India

**Keywords:** fertilizer requirements, target yield, QUEFTS, SSNM, wheat production

## Abstract

Crop modeling can provide us with information about fertilizer dose to achieve the target yield, crop conditions, etc. Due to conventional and imbalanced fertilizer application, nutrient use efficiency in wheat is low. Estimation of fertilizer requirements based on quantitative approaches can assist in improving yields and nutrient use efficiency. Field experiments were conducted at 20 sites in eastern India (Nadia district of West Bengal) to assess the soil supply, requirement, and internal efficiency of N, P, K, and Zn in wheat. The data were used to calibrate the QUEFTS (Quantitative Evaluation of the Fertility of Tropical Soils) model for site-specific, balanced fertilizer recommendations. The parameters of maximum accumulation (a) and maximum dilution (d) in wheat were calculated for N (35, 100), P (129, 738), K (17, 56), and Zn (21502, 140244). Grain yield of wheat showed statistically significant correlation with N (R^2^ = 0.937**), P (R^2^ = 0.901**), and K uptake (R^2^ = 0.801**). The NPK ratio to produce 1 tonne grain yield of wheat was calculated to be 4.9:1.0:8.9. The relationships between chemical properties and nutrient-supplying capacity of soils were also established. The model was validated using the data from four other experiments. Observed yields with different amounts of N, P, K, and Zn were in good agreement with the predicted values, suggesting that the validated QUEFTS model can be used for site-specific nutrient management of wheat.

### INTRODUCTION

Due to conventional blanket and imbalanced fertilizer application, nutrient use efficiency in wheat is low. The site-specific nutrient management (SSNM) strategies that include crop nutrient requirement, indigenous nutrient supply, and recovery efficiency of applied fertilizer should be used to increase yield of wheat. Estimates of indigenous nutrient supply, nutrient requirements, internal efficiency, recovery efficiency of nutrients, and subsequent fertilizer recommendations for wheat (*Triticum aestivum* L.) have been made through field experiments at several researcher sites. However, these estimates can only partly be extrapolated to farmers' fields because of the much broader range of soil, climatic, and management conditions. Therefore, estimates of fertilizer requirements should be based on more generic, quantitative approaches, such as the use of simulation models. Most existing models address only a single nutrient and interaction of the nutrients is largely ignored.

The crop removal of nutrients per unit area of cultivated land are increased considerably[1]. There are uncertainties about N, P, and K requirements of wheat because the internal nutrient efficiencies (IE) vary greatly depending on nutrient supply, crop management practices, and climatic conditions[2]. The new technologies like computer-based crop modeling can provide us a vast amount of real-time information about fertilizer dose to achieve the target yield, crop conditions, weather, etc. The models can be easily used by the farmers, which would enable them to make more precise application of inputs.

The QUEFTS (Quantitative Evaluation of the Fertility of Tropical Soils) model, originally developed by Janssen et al.[3], and further evaluated by others[4,5] is used to process the user's data and the exterior data into personalized information or decision support system (DSS). The QUEFTS also enhances understanding of data taken under certain conditions and helps to extrapolate their applications to other locations. This model facilitates a better understanding of the interrelationship between soil or crop management and various components in a system and can integrate numerous experimental results from different conditions. Integration of this simulation model with agricultural field research may be the best way to bring about further improvements. The QUEFTS model can also be used to help assess different crops and nutrients. Keeping these in view, the present investigation was undertaken for fertilizer requirements of wheat with the following objectives: (1) to estimate N, P, and K requirements; (2) to estimate indigenous nutrient-supplying capacity of the soil; (3) to determine recovery efficiency of nutrients; and (4) to evaluate SSNM in wheat.

### MATERIALS AND METHODS

#### Determinations of Use Efficiencies

The following procedures were used to describe nutrient use efficiencies:

$$RENu = (UNu - Unu_0)/FNu \quad \ldots\ldots\ldots\ldots\ldots\ldots\ldots\ldots\ldots\ldots\ldots (1)$$

where RE is apparent recovery efficiency of applied fertilizer nutrient (kg nutrient in plant dry matter per kg nutrient applied), Nu is the nutrient of concern, UNu is plant nutrient accumulation in total above-ground plant dry matter at maturity (kg/ha) in plots receiving the respective fertilizer nutrient at the rate of FNu (kg/ha), and $UNu_0$ is the total nutrient accumulation without nutrient addition[6].

$$IEx = Y/UNu \quad \ldots\ldots\ldots\ldots\ldots\ldots\ldots\ldots\ldots\ldots\ldots\ldots\ldots\ldots (2)$$

where IEx is internal nutrient efficiency (kg grain per kg nutrient in plant dry matter) and Y is grain yield (kg/ha)[5].

#### Trial Details

Two trials were used in this experiment. The first one was for the determination of maximum accumulation (a) and maximum dilution (d) values for QUEFTS. The second trial was conducted for SSNM to compare observed and simulated values of yield and nutrient uptake where the fertilizer requirement of different nutrients were fixed based on the QUEFTS model to achieve the target yields of 5 and 6 t/ha. The first and second trials comprised of 20 plots in 4 sites (farmers' fields), respectively.

### Model Background

Wheat yields in some places are showing a declining trend. This may be due to imbalanced use of fertilizers that not only reduce the fertilizer use efficiency, but also enhance soil nutrient depletion resulting in a net decrease in the crop yield[7,8]. Estimation of fertilizer requirements based on quantitative approaches can assist in improving wheat yields and increasing nutrient use efficiency. We used the QUEFTS model for estimation of nitrogen (N), phosphorus (P), potassium (K), and zinc (Zn) requirements and their recommendations for a target yield (5 and 6 t/ha) of wheat. Although the original version of QUEFTS was written for maize, it was reprogrammed by C. Witt at the International Rice Research Institute, Philippines for rice. The same program (modified by C. Witt) of QUEFTS was used in this investigation. The model considers the interactions of N, P, K, and Zn and climate-adjusted potential yield of the concerned areas or regions.

A field-tested model (QUEFTS) can be used to transfer the results of experimental research to other soils, climate, and management conditions outside the experimental sites. QUEFTS assumes that yield is a function of N, P, and K supply from soil and fertilizer. In the model, a distinction is made between potential supply of a nutrient (maximum quantity that is supplied from soil and fertilizer) and its actual uptake by the crop. Actual uptake of a nutrient equals potential supply only if all other growth conditions are optimal. The essence of QUEFTS is the relation between nutrient uptake and yield. The model is also used for the nutrients other than N, P, and K.

### Steps of QUEFTS

QUEFTS involves four steps:

- Step I. Assessment of potential indigenous nutrient supply. We used nutrient uptake in fertilizer omission plots as a measure of soil nutrient supply and established relationships with soil chemical tests.
- Step II. Estimation of uptake of N (UN), P (UP), and K (UK) as fractions of potential supply of N (SN), P (SP), and K (SK), i.e., supply from soil plus fertilizer, taking recovery efficiency of applied nutrient into account.
- Step III. Designation of yield ranges as functions of actual uptakes of N, P, and K when they are maximally accumulated and diluted.
- Step IV. Calculation of the final yield estimate by combining the yield ranges for nutrients by accounting of their interactions.

### Determination of "a" and "d" Values for QUEFTS [Experiment I]

Maximum accumulation (a) and maximum dilution (d) values of N, P, K, and Zn on the basis of internal efficiencies were determined for wheat (cv. UP-262) growing in an Inceptisol soil. The experiments were conducted at 20 sites [pH, 6.80–7.65; organic C (%), 0.41–0.56; available N (kg/ha), 469.36–542.25; available P (kg/ha), 9.50–18.75; available K (kg/ha), 59.00–102.50, and available Zn (mg/kg), 0.44–0.67]. The experimental site in the Nadia district in West Bengal is situated at about 130 km North of Bay of Bengal, India and the experiment took place during the years 2001–2002 and 2002–2003 in a randomized block design. There were seven treatments such as: $T_1$, -N; $T_2$, -P; $T_3$, -K; $T_4$, -Zn; $T_5$, +NPKZn; $T_6$, FYM only; and $T_7$, and control.

The recommended dose of N, P, K, Zn, and FYM for wheat were 100, 50, 50, 0.5 kg/ha, and 6 mg/ha, respectively. The yield and uptake data were recorded for the plots including the nutrient omission plot of N, P, K, and Zn. In QUEFTS model, "a" and "d" values were calculated by slope of envelope function grain yield vs. nutrient uptake. The envelope lines of nutrient accumulation and dilution were determined on the basis of internal efficiency of different nutrients which were further used as inputs in QUEFTS. Three sets of constants of "a" and "d" were estimated by excluding the upper and lower 10$^{th}$ percentile (Set I), 15$^{th}$ percentile (Set II), and 20$^{th}$ percentile (Set III) of calculated internal nutrient efficiencies. The 20$^{th}$ percentile as outlier means exclusion of 20% of total data, where 10% of total data has been excluded from the upper and the remaining 10% of total data from lower part. Thus, only 20% of total data were excluded and remaining 80% was retained. Those derived three sets of "a" and "d" values of QUEFTS model were used in wheat for fertilizer recommendations and simulation of nutrient uptake.

### Interpretation of Data

The following relationships were developed: (1) relationship between supply of nutrients (nutrient uptake in omission plot) and available nutrients of N, P, K, and Zn in postharvest soils of wheat were established by developing equations; (2) yield of wheat in relation to plant nutrients of N, P, and K at 5, 6, 7, and 8 t/ha potential yields were estimated through the use of QUEFTS model; and (3) comparison of observed and simulated (using QUEFTS) nutrient uptake of N, P, and K by wheat was made.

### Model Validation

The data from five field studies previously conducted by different scientists at various locations in India were chosen to validate the QUEFTS model. The predicted yield was calculated based on yield and nutrient uptake data.

### Application of the Model for SSNM in Wheat [Experiment II]

Field experiments on wheat (*Triticum aestivum* L.) cv. UP-262 were conducted in the *rabi* season of 2002–2003 in a farmer's field, (the block area of Ranaghat II, district of Nadia of West Bengal [22°57′ N latitude and 88°20′ E longitude, average altitude of 7.8 m above sea level]) with four replications in a randomized block design. The physicochemical properties of the four sites were pH, 7.15–7.33; organic C (%), 0.42–0.46; available N (kg/ha), 441.20–508.08; available P (kg/ha), 8.40–14.25; available K (kg/ha), 77.00–92.80; and available Zn (mg/kg), 0.39–0.55. Seeds were sown at 100 kg/ha. The recommended levels of N, P, K, Zn, and FYM for wheat were 100, 50, 50, 0.5 kg/ha with suitable carrier and 6 t/ha, respectively. Each field was divided into six subplots using the following treatments: $T_1$: Set I, target yield of 5 t/ha; $T_2$: Set I, target yield of 6 t/ha; $T_3$: Set II, target yield of 5 t/ha; $T_4$: Set II, target yield of 6 t/ha; $T_5$: Set III, target yield of 5 t/ha; and $T_6$: Set III, target yield of 6 t/ha. Fertilizer dose of N, P, and K was fixed on the basis of the QUEFTS model by putting various "a" and "d" values of N, P, and K of Set I, Set II, and Set III for achieving the target yield of 5 and 6 t/ha in wheat. Observed yield and uptake by wheat were compared with target yields and predicted or simulated (using QUEFTS) nutrient uptake. The best set of "a" and "d" values of QUEFTS was selected based on potential yield and validation.

### Climatic Conditions

The research station has a humid subtropical climate. During the rice-growing season of 2001–2002 and 2002–2003, the total rainfall and average maximum and minimum temperature were 954.90 and 840.10 mm; 32.55 and 32.94°C; 25.48 and 24.75°C, respectively. Similarly during the wheat-growing season of 2001–2002 and 2002–2003, the corresponding figures were 39.2 and 142.41 mm; 29.32 and 28.24°C; 15.85 and 16.43°C, respectively. The data were collected from the nearest Meteorological Department of the Bidhan Chandra Krishi Viswavidyalaya. It is valid for all the experimental sites since the 20 sites of experiment I were situated in the close vicinity.

### Soil Analysis

(1) Soil pH was determined in 1:2 soil-water suspension ratio[9]; (2) EC was done in the supernatant liquids of the 1:2 soil water suspension using conductivity meter[9]; (3) organic C was determined by the Walkley and Black method[9]; (4) available N was determined by the Kjeldahl method[9]; (5) available P was determined by the Olsen method[10]; (6) available K was extracted by neutral normal ammonium acetate method[9]; (7) DTPA-extractable Zn was extracted by following the procedure of Lindsay and Norvell[11] and determined with the help of an atomic absorption spectrophotometer (AAS), Perkin Elmer, Model-AAnalyst 100.

### Plant Analysis

Plant samples were determined for N by Kjeldahl distillation as outlined by Jackson[9] after digesting the samples with diacid mixture ($H_2SO_4$:$HClO_4$, 9:1); and P and K by Vanado molybdate blue color and flame photometrically, respectively, as described by Jackson[9] after digesting the samples with ternary acid mixture ($HNO_3$:$HClO_4$:$H_2SO_4$, 10:4:1). Zinc was determined using the method by Jackson[9] with the help of AAS, Perkin Elmer, Model-AAnalyst 100 after digesting the samples with ternary acid mixture ($HNO_3$:$HClO_4$:$H_2SO_4$, 10:4:1).

### Statistical Analysis

All the data of wheat of 2 years were pooled statistically and then the relevant data were statistically analyzed for Duncan’s Multiple Range Test (DMRT), correlation and multiple regression, etc. following the procedures as outlined by Cochran and Cox[12] and Gomez and Gomez[13].

## RESULTS AND DISCUSSION

### Indigenous Supply, Internal Efficiency, N Requirement, and Recovery Efficiency

The following relationship between soil organic carbon (OC) and N uptake by wheat in N-omission plots (Fig. 1) was established for estimation of the soil N supply (SN):

$$\text{SN (kg/ha)} = 103.07\ \text{OC (\%)} + 5.46\ (R^2 = 0.91^{**}) \ldots\ldots\ldots\ldots(3)$$

An alternative to estimate the indigenous N supply could be the grain yield obtained in a non-N plot as grain yield has a good correlation (Y = 0.006 + 35.5, $R^2$ = 0.51**) with N uptake in wheat (Fig. 2). Similar views have also reported for wheat by Pathak et al.[14].

Grain yield ranged from 1.50–5.50 t/ha, with N-application rates varying from 0–100 kg/ha across the sites (Table 1). Total above-ground N accumulation ranged from 21.0–119.5 kg/ha, and an internal efficiency of 32–102 kg grain per kg N, with an average of 62.3 kg grain per kg N. Since the experiments were conducted under irrigated conditions, the variation in internal efficiency was possibly due to variations in the supply of N. Data presented in Table 2 show that to produce 1 tonne of grain, the N requirement varied between 8.3 and 29.6 kg with an average of 17.1 kg.

Recovery efficiency of N (REn) varied between 34.5 and 51.2% (Table 1) with an average of 41.3%. Recovery of applied N varied considerably with the amount of N applied. A close relationship between recovery efficiency and level of N (Fn) was observed, as depicted by the equation.

$$\text{REn (\%)} = (0.55\ \text{Fn} + 7.10)/\text{Fn}\ (R^2 = 0.64^{**}) \ldots\ldots\ldots\ldots(4)$$

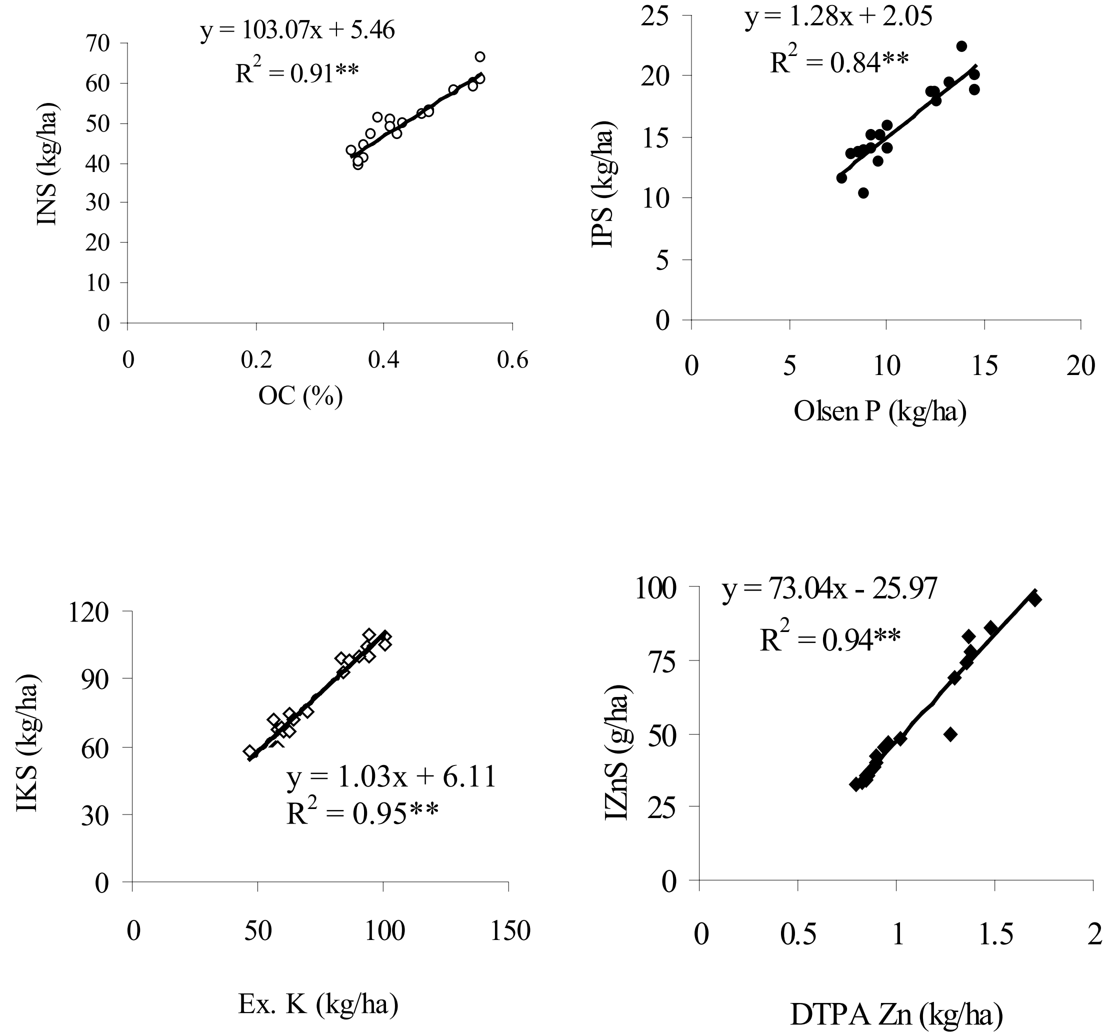

**FIGURE 1.** Relationship between indigenous N, P, K, and Zn with organic C, Olsen P, exchangeable K, and DTPA-Zn, respectively. Data are based on wheat experiments conducted at 20 sites in the Nadia district of West Bengal during the years 2001–2002 and 2002–2003.

This suggested that instead of using a fixed value for recovery efficiency, the above equation can be used for wheat in eastern India where soils are generally low in organic C (<0.5%).

### Indigenous Supply, Internal Efficiency, P Requirement, and Recovery Efficiency

For Indian conditions, the Olsen method is generally used to estimate available P supply of soils with pH values ranging between 6 and 8.5[15]. The following relationship was established between Olsen P in soil and plant P (Fig. 1) to calculate indigenous P supply (SP):

SP (kg/ha) = 1.28 Olsen P (kg/ha) + 2.05 ($R^2$ = 0.84**) …………………(5)

Alternatively, grain yield in P-omission plots can also be used as an index of soil P supply since there was a good correlation and coefficient of determination of variability (Y = 0.009 X – 8.73, $R^2$ = 0.53**) between grain yield and P uptake (Fig. 2).

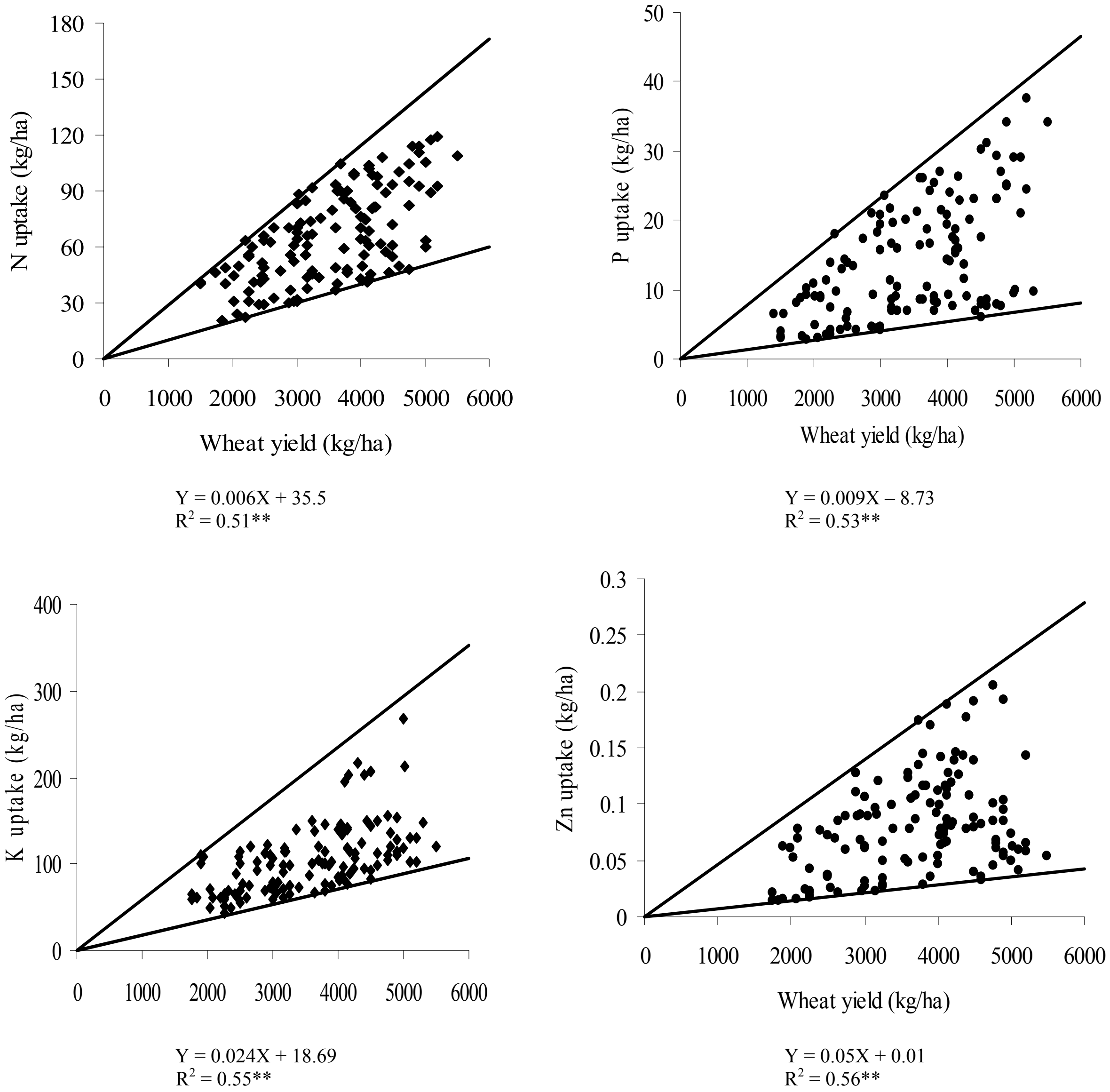

**FIGURE 2.** Relationship between grain yield of wheat and plant nutrients of N, P, K, and Zn. The upper and lower lines indicate yields with maximum dilution and maximum accumulation, respectively, by taking upper and lower 10th percentile as outliers. Although 10% of the total data lie outside the lines, they were not shown in the figure as they are of less importance. Data are based on experiments conducted at 20 sites in the Nadia district of West Bengal during the years 2001–2002 and 2002–2003.

Grain yield of wheat in the experiments with P ranged from 1.40–5.30 t/ha, with application of P varying between 0 and 50 kg/ha (Table 1). Above-ground P accumulation ranged from 2.7–37.5 kg/ha. Internal efficiency of P ranged between 125 and 740 kg/kg, with a mean of 290.4 kg. To produce 1 tonne of wheat grain, 2.1–5.2 kg P was needed with a mean of 3.8 kg P. Recovery efficiency of P varied from 14.2–29.8%, with a mean of 21.2% (Table 1). The following relationship was established between recovery efficiency of P and P fertilizer levels (Fp):

$$REp\ (\%) = [1.58 + 0.33\ Fp - 0.004\ (Fp)^2]/Fp\ (R^2 = 0.71^{**}) \quad \ldots\ldots\ldots\ldots\ldots\ldots(6)$$

**TABLE 1**
**Range of Grain Yield of Wheat, Plant Nutrients, and Fertilizer Application Data Used for Estimation of Internal Efficiency, Plant Requirement, and Nutrient Recovery Efficiency**

| Parameters | Nitrogen | Phosphorus | Potassium | Zinc |
|---|---|---|---|---|
| Yield (t/ha) | 1.50–5.50 (160)[a] | 1.40–5.30 (160) | 1.75–5.50 (160) | 1.75–5.50 (160) |
| Uptake (kg/ha) | 21.0–119.5 (160) | 2.7–37.5 (160) | 43.0–216.0 (160) | 0.01–0.20 (160) |
| Fertilizer applied (kg/ha) | 0–100 (100) | 0–50 (100) | 0–50 (100) | 0–0.5 (100) |
| Available soil nutrient (kg/ha) | 390.4–506.5 (160) | 1.90–16.1 (160) | 60.1–94.2 (160) | 0.18–1.88 (160) |
| Internal efficiency (kg/kg) | 32–102 | 115–740 | 14–59 | 20805–145230 |
| Nutrient requirement (kg/t grain) | 8.3–29.6 | 2.1–5.2 | 13.7–40.1 | — |
| Recovery efficiency (%) | 34.5–51.2 | 14.2–29.8 | 41.1–61.4 | — |
| Estimated parameters | | | | |
| Internal efficiency (kg/kg) | 63.1 | 298.6 | 35.9 | 70322.1 |
| Nutrient requirement (kg/t grain) | 15.8 | 3.2 | 28.4 | — |
| Recovery efficiency (%) | 40 | 20 | 50 | — |

[a] Number of observations in parentheses. Data are based on wheat experiments conducted in 20 sites in Nadia district of West Bengal during the year 2001–2002 and 2002–2003.

**TABLE 2**
**SSNM in Wheat: Effect of Different Treatments Derived from QUEFTS on Observed Grain Yield (t/ha) and Uptake of N, P, and K (kg/ha) by Wheat**

| | SIMULATED | | | | OBSERVED | | | |
|---|---|---|---|---|---|---|---|---|
| Treatments | Target Yield | N Uptake | P Uptake | K Uptake | Grain Yield | N Uptake | P Uptake | K Uptake |
| $T_1$ | 5 | 81.90 | 16.80 | 157.10 | 4.75[d†] | 76.48[d] | 12.05[d] | 151.20[d] |
| $T_2$ | 6 | 106.60 | 21.80 | 204.40 | 5.61[b] | 101.28[a] | 16.85[a] | 199.85[a] |
| $T_3$ | 5 | 78.80 | 15.80 | 142.10 | 4.89[c] | 75.35[d] | 13.18[c] | 139.10[e] |
| $T_4$ | 6 | 99.40 | 20.00 | 179.40 | 5.85[a] | 96.45[b] | 17.33 [a] | 175.25[b] |
| $T_5$ | 5 | 78.30 | 15.70 | 136.80 | 4.60[e] | 72.80[e] | 11.20 [d] | 129.60[f] |
| $T_6$ | 6 | 95.30 | 19.10 | 166.50 | 5.63[b] | 89.88[c] | 15.00 [b] | 158.70[c] |
| SEm (±) | | | | | 0.0230 | 0.3583 | 0.2976 | 0.5295 |

† Within a column, means followed by the same letter are not significantly different at the 0.05 level of probability by Duncan's Multiple Range Test (DMRT).

$T_1$ = NPK @ 89:25:91 kg/ha, $T_2$ = NPK @ 154:52:108 kg/ha, $T_3$ = NPK @ 81:21:109 kg/ha, $T_4$ = NPK @ 135:42:127 kg/ha, $T_5$ = NPK @ 80:20:94 kg/ha, $T_6$ = NPK @ 124:38:110 kg/ha. (These NPK fertilizer doses were fixed based on QUEFTS model.)

$T_1$ and $T_2$ fertilizer doses were fixed based on Set I "a" and "d" values of QUEFTS; $T_3$ and $T_4$ fertilizer doses were fixed based on Set II "a" and "d" values of QUEFTS; $T_5$ and $T_6$ fertilizer doses were fixed based on Set III "a" and "d" values of QUEFTS.

### Indigenous Supply, Internal Efficiency, K Requirement, and Recovery Efficiency

Ammonium acetate-extractable K is widely used as a parameter of available K for wheat also. A relationship (Fig. 1) between K uptake in non-K plots, a measure of soil K supply (SK), and exchangeable soil K (kg/ha) was established:

SK (kg/ha) = 1.03 Ex. K (kg/ha) + 6.11 ($R^2$ = 0.95**) ……………………(7)

Alternatively, soil K supply can also be obtained from the grain yield of K-omission plots, as grain yield has a good correlation (Y = 0.024 X + 18.69, $R^2$ = 0.55**) with K uptake (Fig. 2).

Grain yield of wheat in the experiments with K ranged between 1.75 and 5.50 t/ha and K uptake from 43.0–216.0 kg/ha (Table 1). Internal efficiency of K ranged from 14–59 with a mean of 33.4 kg grain per kg K. To produce 1 tonne of grain, 13.7–40.1 kg K was needed (mean 29.7 kg/t). Recovery of K varied between 41.1 and 61.4 with a mean of 51.2% (Table 1). The following equation was developed that can be used for the recovery efficiency of K (REk) in soils containing medium to higher exchangeable K content with the help of K fertilizer levels (Fk):

REk (%) = [–5.22 + 0.78 Fk – 0.003 $(Fk)^2$]/Fk ($R^2$ = 0.55**) ………………(8)

### Indigenous Supply and Internal Efficiency of Zn

A relationship (Fig. 1) between Zn uptake in non-Zn plots, a measure of soil Zn supply (SZn), and DTPA-extractable Zn in soil (kg/ha) was established (Fig. 1).

SZn (g/ha) = 73.04 DTPA-Zn (kg/ha) – 25.97 ($R^2$ = 0.94**) …………….(9)

An alternative to estimate indigenous Zn supply could be the grain yield obtained in a non-Zn plot as grain yield has a good correlation (Y = 0.05 X + 0.01, $R^2$ = 0.56**) with Zn uptake in wheat (Fig. 2).

Grain yield ranged from 1.75–5.50 t/ha, with Zn application rates varying from 0–0.5 kg/ha with suitable carrier across the sites (Table 1). Total above-ground Zn accumulation ranged from 0.02–0.15 kg/ha, and an internal efficiency of 20805–145230 kg grain per kg Zn with a mean value of 70322.1 kg grain per kg Zn. The estimates of N, P, K, and Zn requirements refer to current crop and fertilizer management practices, but may not reflect the optimum nutritional balance where N, P, K, and Zn are neither limiting nor in surplus. The observed variation in internal efficiencies and nutrient requirements was due to nutritional imbalances. Therefore, a modeling approach is advocated as in QUEFTS, to estimate the optimum nutrient requirements as a basis for an appropriate and improved fertilizer recommendation. Results of a preliminary evaluation are discussed in the following sections.

### Evaluation of “a” and “d” Values

Three sets (Set I, II, and III) of constants of envelope functions relating grain yield to the maximum accumulation (a) and maximum dilution (d) of QUEFTS in irrigated wheat were developed by treating the upper and lower $10^{th}$, $15^{th}$, and $20^{th}$ percentiles of the internal efficiencies as outliers. In order to determine the fertilizer recommendations of N, P, and K to achieve the target yield, these constants were developed. Three sets of values for “a” and “d” were for N (35, 100; 37, 96; and 39, 92), for P (129, 738; 144, 652; and 150, 605), for K (17, 56; 21, 52; and 24, 49) and for Zn (21502, 140244; 24831, 129630; and 27778, 118750), respectively. With an increase in percentiles from the $10^{th}$ to the $20^{th}$, the value of “a” increased while that of “d” decreased markedly. The same trend was observed for all nutrients[14].

### Evaluation of the QUEFTS Model for Wheat in Eastern India

The QUEFTS model was evaluated for wheat using the relations as discussed earlier. The relationships between yield and plant N, P, K, and Zn used were:

$$YNA = 35\ Un \quad \ldots\ldots\ldots\ldots\ldots\ldots\ldots\ldots\ldots(10)$$

$$YND = 100\ Un \quad \ldots\ldots\ldots\ldots\ldots\ldots\ldots\ldots\ldots(11)$$

$$YPA = 129\ Up \quad \ldots\ldots\ldots\ldots\ldots\ldots\ldots\ldots\ldots(12)$$

$$YPD = 738\ Up \quad \ldots\ldots\ldots\ldots\ldots\ldots\ldots\ldots\ldots(13)$$

$$YKA = 17\ Uk \quad \ldots\ldots\ldots\ldots\ldots\ldots\ldots\ldots\ldots(14)$$

$$YKD = 56\ Uk \quad \ldots\ldots\ldots\ldots\ldots\ldots\ldots\ldots\ldots(15)$$

$$YZnA = 21502\ Uzn \quad \ldots\ldots\ldots\ldots\ldots\ldots\ldots(16)$$

$$YZnD = 140244\ Uzn \quad \ldots\ldots\ldots\ldots\ldots\ldots(17)$$

where YNA and YND (YPA and YPD, YKA and YKD, YZnA and YZnD) are yields obtained when N (P, K, and Zn) in the wheat crop was maximally accumulated and diluted, respectively; Un, Up, Uk, and Uzn were uptake of N, P, K, and Zn, respectively. From these equations, yield ranges corresponding with the actual uptake of N, P, K, and Zn are calculated. The relationships between yield and plant N, P, K, and Zn (Fig. 2) were presented.

### Sensitivity of QUEFTS to “a” and “d” Values

The sensitivity of the model to “a” and “d” values was tested using three sets of constants by treating the upper $10^{th}$, $15^{th}$, and $20^{th}$ percentiles of the internal efficiencies as outliers. In order to determine the N, P, and K requirements to achieve a target grain yield (Fig. 3), the potential supply of N, P, and K was set as nonlimiting and the yield potential was set to 7 t/ha. Nutrient requirements calculated by the model were similar for all three sets of constants (Fig. 3), except at yield targets that were close to the yield potential. Such yield targets rarely occur in a farmer’s fields. We propose to use the QUEFTS model parameters of Set I for a standard version of QUEFTS focusing on decision making on fertilizer requirements of wheat, as these include the maximum range of variability.

### Nutrient Requirement vs. Potential Yield

Taking into consideration the season and site, the potential yield of currently grown wheat in eastern India ranges from 5–8 t/ha. The relationship between grain yield and nutrient accumulation of N, P, and K as predicted by QUEFTS was linear at lower yield levels, reflecting a situation where plant growth was mainly limited by nutrient supply (Fig. 4). The model also calculates a decrease in internal efficiencies when target yields are close to yield potential. Thus, it may be more profitable for farmers to maximize nutrient efficiencies by a more balanced nutrition than to aim for higher yield targets with yield levels approaching maximum yields.

Regardless of yield potential, the N:P:K ratio for 1 tonne grain in plants for the linear part of the relationship (up to 80% potential yield) was about 4.9:1:8.9 as calculated by QUEFTS (Fig. 4). To produce 1 tonne grain, 15.8, 3.2, and 28.4 kg N, P, and K, respectively, would be needed with this, internal efficiencies (IE) of 63.1, 298.6, and 35.9 kg grain per kg N, P, and K, respectively, would be achieved. Results also supported the findings obtained by Pathak et al.[14] who suggested that the required N, P, and K accumulation in the wheat plant for 1 tonne grain yield was 23.1, 3.5, and 28.5 kg, respectively, which indicated the NPK ratio in the plant of about 6.6:1:8.1.

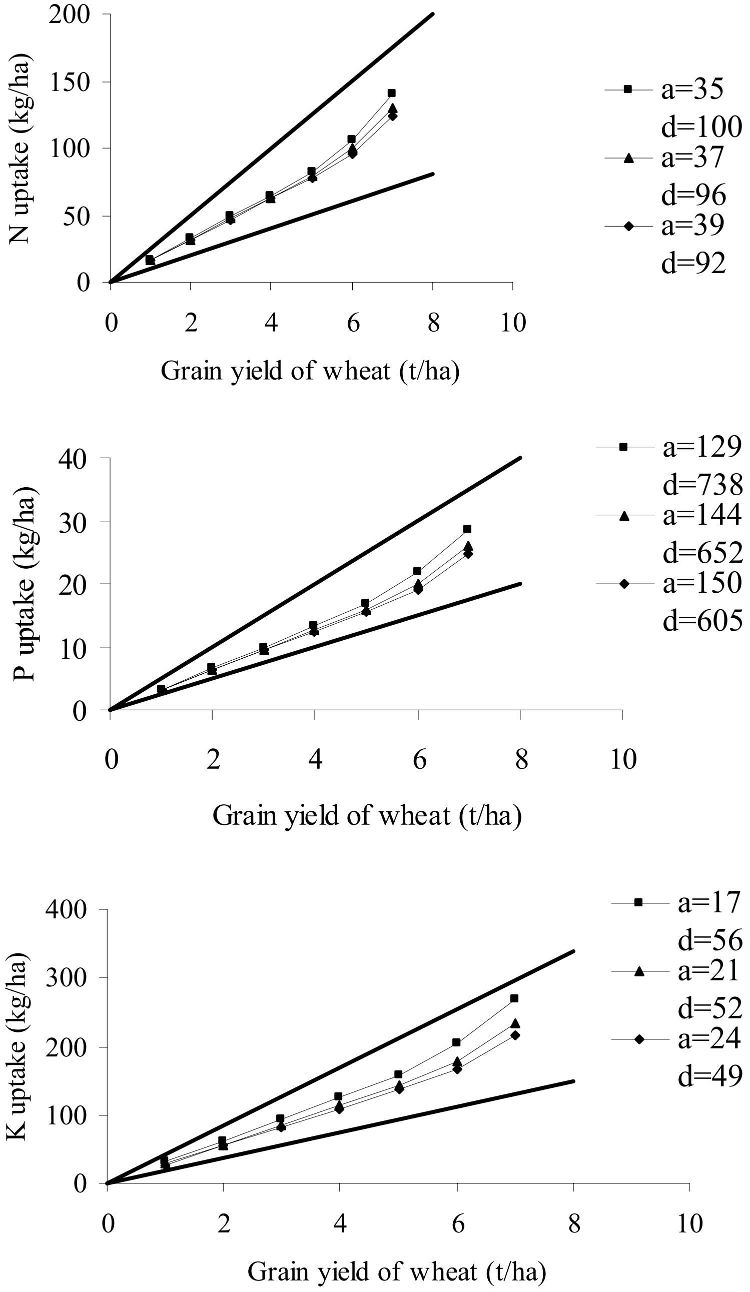

**FIGURE 3.** Yield of wheat in relation to plant nutrients at different sets of constants "a" and "d", calculated by excluding the upper and lower 10th (Set I), 15th (Set II), and 20th percentiles (Set III) of all internal efficiency data. The upper and lower lines indicate yields with maximum dilution and maximum nutrient accumulation, respectively.

### Preliminary QUEFTS Model Validation

The data from five field studies previously conducted by different scientists at the following locations in India were chosen to validate the calibrated model: (1) three villages around Delhi (28.35° N, 77.12° E)[16], (2) Indian Agricultural Research Institute, New Delhi (28.55° N, 77.52° E)[17], (3) Hisar, Haryana (29.10°

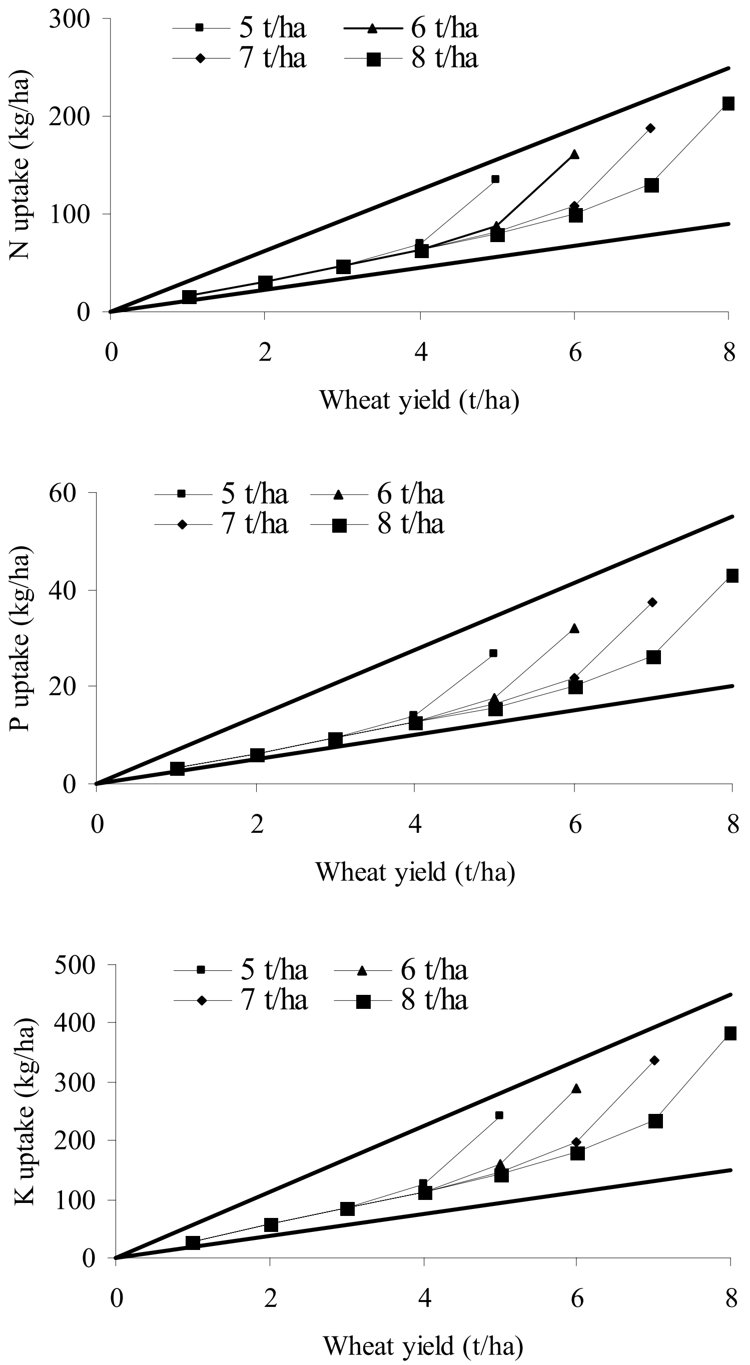

**FIGURE 4.** Yield of wheat in relation to plant nutrients at 5, 6, 7, and 8 t/ha potential yields. The upper and lower lines indicate yields with maximum dilution and maximum nutrient accumulation, respectively.

N, 75.46° E)[18], (4) Udaipur, Rajasthan (24.35° N, 73.42° E)[19], and (5) Pantnagar, Uttaranchal (29.00° N, 79.30° E)[20]. Observed yields were in good agreement with the predicted values (Fig. 5), indicating that the model can be used to improve fertilizer recommendations of wheat in India.

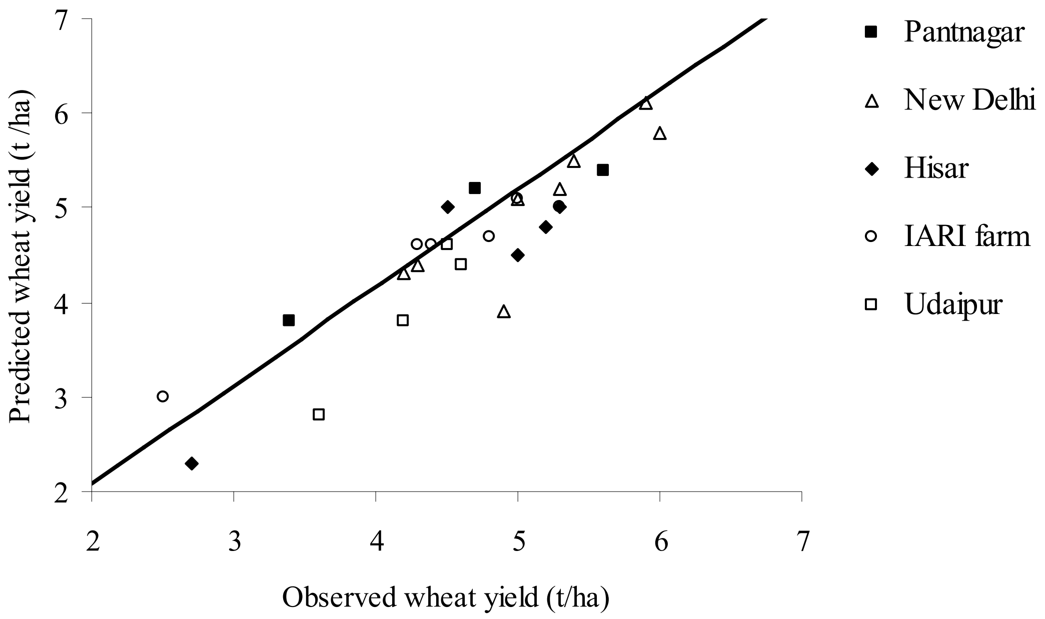

**FIGURE 5.** Yields of wheat predicted by the model QUEFTS and observed in different locations in India.

### SSNM in Wheat

Three sets of "a" and "d" values were used to achieve the target yield to tune of 5 and 6 t/ha wheat. On the basis of QUEFTS, the simulated N, P, and K uptakes were calculated for all sets to achieve the target yield of 5 and 6 t/ha. The simulated N, P, and K uptakes (kg/ha) were compared with their corresponding observed values (Table 2).

The wheat grain yield increased significantly with an increasing level of N, P, and K fertilizers. The highest grain yield (5.85 t/ha) was observed in the treatment $T_4$ where NPK was applied at 135:42:127 kg/ha to achieve the target yield of 6 t/ha. The highest observed N (101.28 kg/ha) and K uptakes (199.85 kg/ha) were found in the treatment $T_2$ where NPK was applied at 154:52:108 kg/ha based on QUEFTS followed by $T_4$, $T_6$, and $T_1$ treatments. The observed N, P, and K uptakes were close (80–90%) to their corresponding simulated or predicted values. The results suggested that there was a need for revision of recommended fertilizer doses for wheat in eastern India to increase the yield level. Ray et al.[21] also suggested that the yield targets were attained for wheat (cv. Sonalika) 30–35 q/ha with ± 10% variation from the desired yield target.

The results (Table 3) indicated that the uptake of N, P, K, and grain yield have been found to be significantly correlated. The grain yield showed a significant correlation with N (0.937**), P (0.901**), and K (0.801**) uptakes. The grain yield of wheat significantly varied with the uptake of N, P, and K and which altogether contributed 95.2% of the variability towards the grain yield of wheat.

### CONCLUSIONS

The results for the use of the QUEFTS model at 20 sites of the alluvial soils (Inceptisols) of the Nadia district of West Bengal show that the model might be applicable for estimating nutrient requirements to achieve a yield target of 5 and 6 t/ha for wheat. The QUEFTS model takes into account the soil nutrient supply, relationship of grain yield vs. nutrient uptake, and balanced uptake of nutrients. The developed parameters of "a" and "d" of N, P, K, and Zn in plants can also be used as standard parameters in the model for wheat. To produce 1 tonne grain yield wheat, the derived NPK ratio was 4.9:1:8.9. The model can also be

**TABLE 3**
**Simple Correlation Matrix Showing the Relationship of Observed N, P, and K Uptake by Wheat with Grain Yield**

| Parameter | N Uptake | P Uptake | K Uptake | Grain Yield |
|---|---|---|---|---|
| N uptake | 1.000 | | | |
| P uptake | 0.900** | 1.000 | | |
| K uptake | 0.947** | 0.804** | 1.000 | |
| Grain yield | 0.937** | 0.901** | 0.801** | 1.000 |

** Significant at the 0.01 probability level.

used in other regions, provided that appropriate equations between soil's ability to supply and chemical properties are determined. The observed yields of wheat with different amounts of these nutrients were in good agreement with the values predicted by the QUEFTS model.

### ACKNOWLEDGMENTS

The authors are grateful to the Indian Council of Agricultural Research (ICAR), New Delhi for sponsoring this National Agricultural Technology Project (ICAR/NATP/R-W/7B-3, PSR-25) which was successfully completed in collaboration with International Rice Research Institute (IRRI), Philippines and Rice-Wheat Consortium (RWC), New Delhi. It is beyond our grasp of words to acknowledge the farmers of Dhantala and Nokari, District of Nadia, West Bengal for providing their land for conducting farmer's field experiments.
